# Gintonin enhances epithelial barrier function by activating NRF2 pathway in radiation-induced intestinal injury

**DOI:** 10.1016/j.jgr.2025.01.003

**Published:** 2025-01-19

**Authors:** Hyosun Jang, Hyewon Kim, Su-Hyun Oh, Yeonghoon Son, Rami Lee, Seung-Yeol Nah, Hae-June Lee

**Affiliations:** a Laboratory of Radiation Exposure & Therapeutics, Korea Institute of Radiological & Medical Sciences, Seoul, Republic of Korea; b Division of Radiation Biomedical Research, Korea Institute of Radiological & Medical Sciences, Seoul, Republic of Korea; c Ginsentology Research Laboratory and Department of Physiology, College of Veterinary Medicine, Konkuk University, Seoul, Republic of Korea

**Keywords:** Gintonin, Radiation-induced intestinal injury, NRF2, Epithelial barrier

## Abstract

**Background:**

Because the intestine is a radio-sensitive organ in the body, and radiation-induced intestinal injury is a major clinical problem associated with radiotherapy or radiological accidents. Dysfunction of the epithelial barrier leads to bacterial translocation to other organs, resulting in severe inflammation. Recent findings suggest that gintonin (GT) suppresses oxidative stress and inflammation in neuroinflammatory diseases.

**Purpose:**

This study objected to elucidate the mitigating effects of GT on radiation-induced intestinal injury.

**Methods:**

The therapeutic effects of GT were assessed in a mouse model of radiation-induced intestinal injury using histological, immunohistochemical, and real-time PCR. Additionally, the direct effects of GT and NF-E2-related factor 2 (NRF2) activators on radiation-induced epithelial damage were assessed using Caco-2 cell monolayers.

**Results:**

GT treatment reversed radiation-induced body weight loss, attenuated intestinal damage, and inhibited inflammatory response by reducing inflammatory cell infiltration and cytokine expression in the intestines of mice. Additionally, GT treatment activated NRF2 and ameliorated intestinal barrier damage. In vitro experiments showed that GT treatment affected epithelial permeability and intercellular junction expression in Caco-2 cell monolayers under irradiated conditions. Moreover, treatment with NRF2 activator improved epithelial permeability, improved the expression of intercellular junctions in irradiated epithelial cells, and attenuated radiation-induced intestinal injury in a mouse model.

**Conclusion:**

GT maintains epithelial integrity by activating NRF2-mediated antioxidant activity in radiation-induced intestinal epithelial damage of mice. Overall, these results suggest that GT could be a novel therapeutic agent for radiation-induced intestinal damage.

## Graphical abstract

**Figure:**
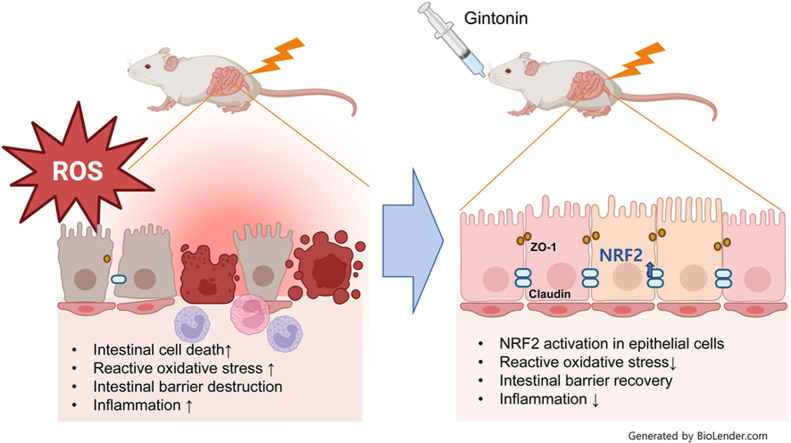

## 1 Introduction

The intestinal epithelium is characterized by rapid and continuous proliferation and regeneration, and the gastrointestinal (GI) tract is radiation-sensitive [1]. Particularly, radiation-induced intestinal damage is an important clinical problem in radiotherapy-received patients and nuclear accident or radiological terrorism victims [2]. Radiation-induced intestinal toxicity manifests as vomiting, diarrhea, dehydration, intestinal and systemic inflammation, infection, endotoxemia, and death [3]. These clinical signs may lead to a reduced quality of life for the patient and necessitate a decrease in radiation therapeutic dose, which may limit the success of radiation therapy. Although several countermeasures for hematopoietic damage already have the US food and Drug Administration (FDA) investigational new drug status (CBLB502, 5-AED, Bio300, EX-Rad) are in development, there are still no FDA-approved effective mitigators for the radiation-induced intestinal injury at present. Therefore, the optimization of radiation therapy techniques and pharmacological interventions is necessary to attenuate acute and chronic GI damage that may occur during radiation therapy.

The intestinal epithelium, also known as the barrier, is a single layer of epithelial cells found in the gut lumen. The intestinal barrier not only prevents the influx of external pathogens and toxins but also plays an important role in selective permeability to allow the passage of nutrients, electrolytes, and water from the gut lumen into the blood [4]. In the intestinal epithelium, intercellular junctions, including adherent junctions (e.g., E-cadherin), tight junctions [TJs; e.g., zonula occludens 1 (ZO1), claudin 3 (CLDN3), and occludin), and desmosomes (e.g., desmocolin and desmoglein 2 (DSG2)], are concerned with the maintenance of intestinal epithelial barrier function. TJs are composed of both transmembrane and extrinsic membrane proteins that control paracellular permeability and maintain the epithelial barrier function. The loss of TJs results in epithelial barrier dysfunction, leading to bacterial translocation to other organs and excessive inflammatory reactions [5,6]. Several patients receiving abdominal radiotherapy progress to intestinal inflammation caused by the disturbance of epithelial homeostasis and the epithelial barrier within a few weeks [7]. Recently, TJs have been reported as important therapeutic targets for radiation-induced intestinal injury [4,8].

Oxidative stress induces intestinal barrier dysfunction with epithelial TJ loss [9] and inflammation [9,10]. Oxidative stress plays a critical role in the early stages of intestinal diseases and pathogenesis of radiation-induced intestinal injury. Radiation exposure induces reactive oxygen species (ROS)-derived oxidative stress and exacerbates inflammation and epithelial homeostasis. NF-E2-related factor 2 (NRF2) is a transcription factor and modulates oxidative stress-induced damage. For example, NRF2 deficient mouse model are sensitive to drug-induced toxicity, oxidative stress-induced inflammation, and radiation-induced tissue injury [[11], [12], [13]]. NRF2 suppresses inflammation by regulating the transcription of pro-inflammatory cytokine genes [14]. Moreover, NRF2 dependent pathway upregulates epithelial TJ proteins [15].

Gintonin (GT) is a non-saponin component of Panax ginseng that consists of a group of glycolipoproteins containing large amounts of lysophosphatidic acid (LPA), carbohydrates, and hydrophobic amino acids [16]. LPA (1-acyl-2-hydroxy-sn-glycero-3-phosphate) serves as the active component of gintonin, which acts as an exogenous ginseng-derived ligand for G protein-coupled LPA receptors [16,17]. This characteristic sets gintonin apart from other known ginseng compounds, such as saponins and acidic polysaccharides, as its primary functional element is a lipid ligand derived from ginseng [17]. GT has been reported to exert therapeutic effects in neuroinflammatory diseases, such as Parkinson's disease [18] and Alzheimer's disease [17,19,20], arthritis [21,22], atopic dermatitis [23], and multiple sclerosis [24,25] by regulating oxidative stress and inflammation. However, the mitigative effects of GT on radiation-induced intestinal damage have not been explored. Therefore, this study aimed to investigate the protective effects of GT in radiation-induced intestinal barrier damage and assess its therapeutic effects on radiation-induced intestinal damage.

## 2 Material and methods

### 2.1 Mice

C57BL/6 mice (male, 6-weeks old) were puechased from DooYeol Biotech (Seocho-gu, Seoul, Korea) and sustained under specific pathogen-free conditions (21 ± 2 °C,12-h light/dark cycle) in the animal facility at the Korea Institute of Radiological and Medical Sciences (KIRAMS). After adaptation for seven days, the mice were randomly divided into experimental groups. All experimental procedures were performed in accordance with the guidelines of the IACUC of KIRAMS (KIRAMS 2023–0087).

### 2.2 Irradiation and treatment with GT and NRF2 activator

Briefly, the mice were anesthetized with a combined injection of alfaxalone (85 mg/kg, Alfaxan®; Careside, Gyeonggi-do, Korea) and xylazine (10 mg/kg, Rompun®; Bayer Korea, Seoul, Korea), followed by irradiation of the whole abdomen with a 13.5 Gy (2.0 Gy/min) using a X-ray irradiator (X-RAD 320, East Haven, CT, USA). After irradiation, mice were daily treated with GT (250 mg/kg/day) or NRF2 activator (CDDO-Im, 1 mg/kg/day; R&D Systems, Minneapolis, MN, USA). The control group in GT experiment was injected with an equal amount of saline. The control group in CDDO-Im experiment was injected with an equal amount of vehicle (5 % DMSO, 5 % Cremophre in saline). On day 6, the mice were euthanized, and intestinal tissue sampling was performed.

### 2.3 Histological analysis of the small intestine

Small intestinal sample of mice were fixed with 10 % neutral buffered formalin solution, embedded with paraffin wax, and cutted 4 μm thickness transversely. The slides were stained with hematoxylin and eosin (H&E). For determination of crypt numbers ∼3–5 field of view per mouse/section were assessed. Villous length was determined by measuring the length from the crypt villus junction to the villous tip. 25–30 numbers of villus assessed per mouse. Histological analysis were quantified in the H&E-stained intestinal slides. The histological score of radiation-induced intestinal injury was evaluated based on 4 categories, including the severity of epithelial damage (0; normal, 1; villi shortening and epithelial loss <10 %, 2; villi shortening and epithelial loss <30 %, 3; villi shortening and epithelial loss >30 %), crypt destruction (0; normal, 1; crypt loss <20 %, 2; crypt loss <50 %, 3; crypt loss >50 %), vascular dysfunction (0; normal, 1; mild enlargement, 2; moderate enlargement, 3; severe enlargement), and inflammatory reaction in the mucosa (0; normal, 1; mild infiltration, 2; moderate infiltration, 3; severe infiltration). For immunohistochemical stain, the slides were incubated with antigen retrieval buffer and 0.3 % hydrogen peroxide in methyl alcohol for 20 min. After PBS washing, the slides were treated with a blocking solution (Vector ABC Elite kit; Vector Laboratories, Burlingame, CA, USA) and incubated with primary antibodies [anti-Ki-67 (Acris), 8-Hydroxy-2-deoxyguanosine (8-OHdG; Abcam), NRF2 (Santa Cruz), CD68 (Abcam), Villin and Zo1 (Invitrogen, Carlsbad, CA, USA), and Cldn3]. And the slides were incubated with horseradish peroxidase-conjugated secondary antibodies (Dako, Carpinteria, CA, USA). The slides were reacted with a diaminobenzidine substrate (Dako) for peroxidase reaction and counterstained with hematoxylin.

### 2.4 Fluorescein isothiocyanate (FITC)-dextran absorption assay in vivo

At the 3 d after irradiation, we performed a midline laparotomy in the anesthetized mice and injected 100 μl FITC-dextran solution (12.5 mg, 4 kDa, Sigma, St Louis, MO) in 5 cm segment of ileum obstructed by bulldog clamp. Subsequently, blood sample was obtained via cardiac puncture 30 min after intraluminal injection and serum separating tubes contained blood sample were centrifuged at 1000×*g* for 15 min to collect serum. The FITC concentration in serum samples was assessed using a fluorescence spectrophotometer (excitation,485 nm; absorption, 528 nm).

### 2.5 Bacterial translocation assay

To determine bacterial translocation ability from the intestinal lumen to the mesenteric lymph nodes, lymph nodes located between cecum and ileum were collected from the mice on day 6 after irradiation. 100 μl supernatant of the lymph node homogenate was treated on MacConkey agar (BD Biosciences, Palo Alto, CA, USA) and cultivated at 37 °C for 18 h. The percentage of colony-positive plates in each group was counted.

### 2.6 Quantitative real-time PCR

Total RNA was extracted from the small intestine and Caco2 monolayers using the TRIzol reagent (Invitrogen, Carlsbad, CA, USA), followed by cDNA synthesis. The expression level of each target gene was normalized to that of internal control (GAPDH). Cycle threshold levels were used to evaluate the relative mRNA levels using the 2^-ΔΔCt^ method. The primer sequences were shown to Table 1.

**Table 1:** Primer sequences used in experiments**.**

Species	Primer	Forward (5′-3′)	Reverse (5′-3′)
Mouse	*Nrf2*	ACACGGTCCACAGCTCATC	TGTCAATCAAATCCATGTCCTG
	*Ho1*	CTCAACATCCAGCTCTTTGAG	AATCTTGCACTTTGTTGCTGGC
	*Nqo1*	GACATCACAGGTAAACTGAAGG	GCAGGGGGAACTGGAATATC
	*Il-6*	AGCCAGAGTCCTTCAGAGAG	GATGGTCTTGGTCCTTAGCC
	*Tnf-a*	AGGGTCTGGGCCATAGAACT	CACCACGCTCTTCTGTCTACT
	*Pai1*	AGGATCGAGGTAAACGAGAGC	GCGGGCTGAGATGACAAA
	*Villin*	CACCTTTGGAAGCTTCTTCG	CTCTCGTTGCCTTGAACCTC
	*Zo1*	AGGACACCAAAGCATGTGAG	GGCATTCCTGCTGGTTACA
	*Cldn3*	AAGCCGAATGGACAAAGAA	CTGGCAAGTAGCTGCAGTG
	*Gapdh*	CATGGCCTCCAAGCAAG	TGTGAGGGAGATGCTCAGTG
Human	*ZO1*	ATCCCTCAAGGAGCCATTC	CACTTGTTTTGCCAGGTTTTA
	*E-cadherin*	GGTTTTCTACAGCATCACCG	GCTTCCCCATTTGATGATGACAC
	*DSG2*	TGGACACCCAAACAGTGGCCCT	CTCACTTTGTTGCAGCAGCACAC
	*HO1*	CTCAACATCCAGCTCTTTGAG	AATCTTGCACTTTGTTGCTGGC
	*NQO1*	GACATCACAGGTAAACTGAAGG	GCAGGGGGAACTGGAATATC
	*GAPDH*	ACCACAGTCCATGCCATCAC	TCCACCACCCTGTTGCTGTA

### 2.7 Cell culture

Caco-2 cells were utilized as the intestinal epithelium in vitro model. Caco-2 cells were maintained in Dulbecco's modified Eagle's medium (Gibco, Grand Island, NY, USA) supplemented with 10 % fetal bovine serum (Gibco) and 1 % penicillin/streptomycin (Gibco) at 37 °C in a 5 % CO_2_ condition. Confluent Caco-2 cell monolayers were used as the intestinal epithelium in vitro model.

### 2.8 Radiation exposure and treatment in vitro

Caco-2 cells were incubated for 5 d to allow for the expression of differentiated epithelium. Thereafter, the cells were irradiated to 15 Gy (3.81 Gy/min) using a^137^Cs gamma-ray source (Gammacell 3000 Elan, MDS Nordion, Ottawa, Canada), and then immediately treated using GT (1 μg/ml), NRF2 activator (CDDO-Im; 100 nM), and/or NRF2 inhibitor (ML385; 10 μM) for 48 h.

### 2.9 Transepithelial electrical resistance (TEER) measurement

The TEER values of the monolayered epithelium were measured using An EVOM system (WPI, Sarasota, FL, USA). Briefly, 1 × 10^6^ Caco-2 cells were spread onto 0.4 μm pore sized 12 Transwell plate (Corning, NY, USA) to form confluent epithelium. After 21 d, the monolayers were irradiated and then handled with GT (1 μg/ml), CDDO-Im (100 nM), and/or ML385 (10 μM). After 48 h, TEER levels were measured.

### 2.10 FITC-dextran assay

Caco-2 cells were spread onto 0.4 μm pore sized 12 transwell (Corning) and maintained for 21 d. After radiation exposure, the upper chamber of the transwell treated with GT (1 μg/ml) or CDDO-Im (100 nM) were analyzed using 500 μg/ml 4 kDa FITC-dextran (Sigma-Aldrich). The FITC intensity in the lower chambers was analyzed using a fluorescence microplate reader (excitation,485 nm; absorption, 528 nm).

### 2.11 Immunofluorescence assay

Caco-2 cells were fixed in 4 % paraformaldehyde were then permeabilized (0.1 % Triton X-100, 5 % bovine serum albumin) for 1 h at room temperature. The cells were incubated (4 °C) overnight with primary antibodies (NRF2; Santacruz). After a brief washing step, the cells were incubated with Alexa Fluor 488-labeled secondary antibody (Thermo Fisher Scientific) for 1 h at room temperature. During the final step, the cells were incubated with DAPI for counterstaining and then mounted using Vectashield HardSet mounting medium (Vector Laboratories).

### 2.12 Statistical analysis

Data were assessed using the GraphPad Prism software. Data are displayed with average ± SE of the average for in vivo data, and average ± SD of average for in vitro experiment data. Statistical significance was assessed using Student's *t*-test compared with two groups or one-way analysis of variance with Tukey's multiple comparison test compared with multiple groups. Statistical significance was established at *p* < 0.05.

## 3 Results

### 3.1 GT treatment alleviates acute radiation-induced intestinal injury in vivo model

**Fig. 1:**
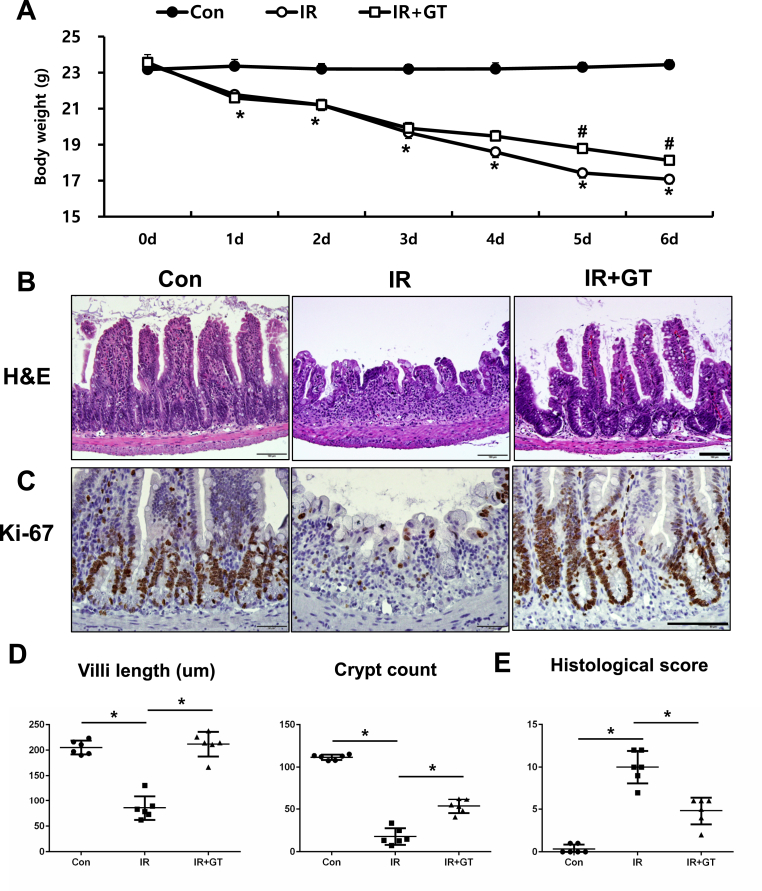
Gintonin (GT) treatment ameliorated acute radiation-induced intestinal injury in a mouse model. (A) Body weight, (B) H&E staining, (C) Immunohistochemical analysis for Ki-67, a proliferation marker, and (D) Villi height and the crypt count in the intestine of the Con, IR, and IR + GT groups at 6 d post treatment. Bar = 100 μm. (E) Histological score determined the levels of epithelial integrity, crypt damage, vascular enlargement, and accumulation of inflammatory cells in the mucosa (each category 0–3 score). Each bar represents mean ± SD of each group of mice (n = 6). ∗*p* < 0.05.

To investigate the mitigating effect of GT on acute radiation-induced intestinal injury, we irradiated the whole abdomen of mice with 13.5 Gy of X-rays and treated them with GT (250 mg/kg/day) orally. Additionally, we monitored body weight changes in irradiated mice treated with or without GT. Severe body weight loss was observed in the IR group (Fig. 1A); however, GT treatment significantly attenuated irradiation-induced weight loss at 5 and 6 d after the treatment (Fig. 1A). Histopathological analysis was performed on the small intestinal tissue at 6 d after irradiation. Crypt destruction, shortening of the villi, loss of the epithelial cells, inflammatory cell accumulation in the mucosa layer, and impaired epithelial proliferation (Ki-67 marker) were observed in the intestines of irradiated mice (Fig. 1B and C). In contrast, villus height and crypt numbers in the small intestine were markedly higher in mice treated with GT after irradiation (IR + GT group) compared to those in the untreated (IR) group (Fig. 1D). Additionally, histological scores, including epithelial damage (0–3), crypt destruction (0–3), endothelial dysfunction (0–3), and inflammatory reaction (0–3) scores, were significantly decreased in the IR + GT group those in the IR group (Fig. 1E). Overall, these results suggest that GT treatment ameliorates acute radiation-induced intestinal damage in a mouse model.

### 3.2 GT treatment reduces oxidative stress and inflammation with NRF2 activation

**Fig. 2:**
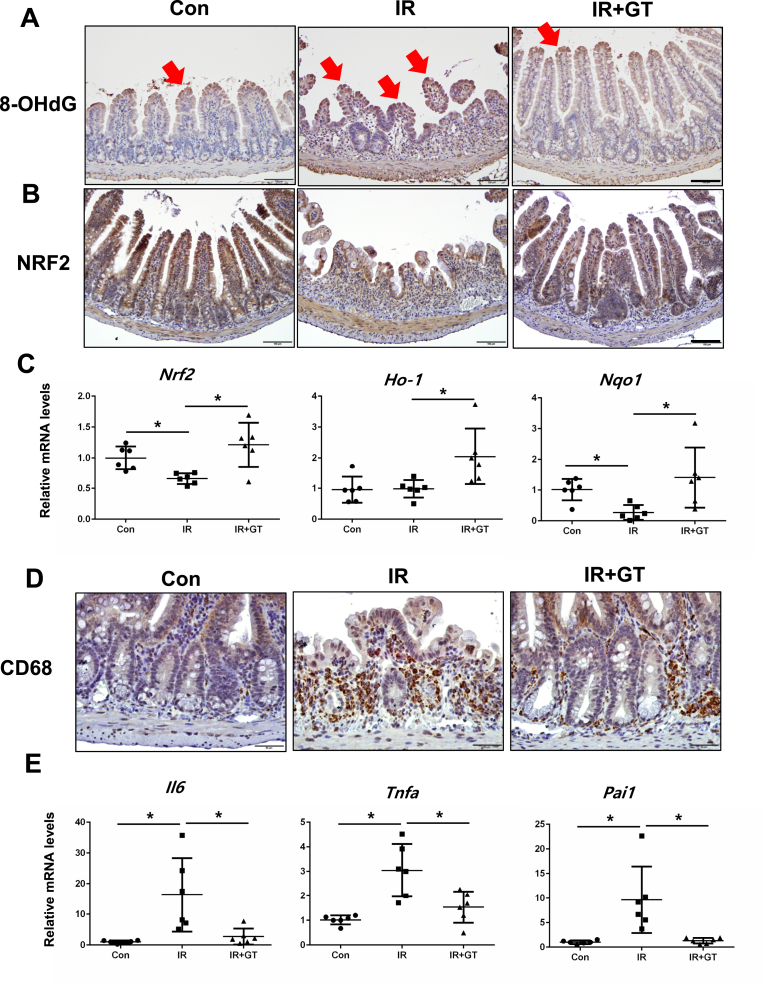
Gintonin (GT) treatment inhibited oxidative stress and inflammation with NRF2 activation. Immunohistochemical analysis for (A) 8-OHdG and (B) Nrf2 expression in the intestine of the Con, IR, and IR + GT groups at 6 d post-treatment. Red arrows specify 8-OHdG positive cells. Bar = 100 μm. (C) Relative mRNA expression of Ho1 and Nqo1 in the intestine. (D) Immunohistochemical analysis for CD68 and (E) mRNA levels of inflammatory cytokines (Il6, Tnf-a, and Pai1) in the intestine of the Con, IR, and IR + GT groups. Bar = 50 μm. Each bar represents mean ± SD of each group of mice (n = 6). ∗*p* < 0.05.

**Fig. 3:**
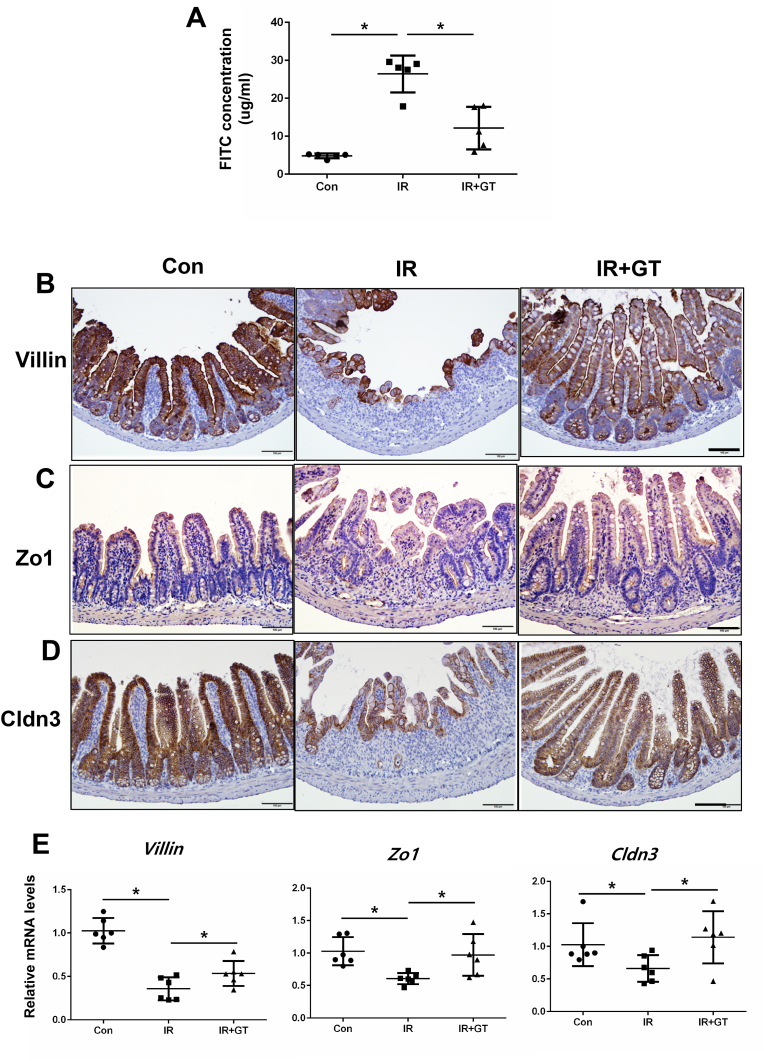
Gintonin (GT) treatment restored radiation-induced barrier destruction in the intestine. (A) Plasma FITC concentration of mice in the Con, IR, and IR + GT groups at 3 d post-treatment (n = 5). Immunohistochemistry for (B) villin, (C) Zo1, and (D) Cldn3. Bar = 100 μm. (E) mRNA levels of villin, Zo1, and Cldn3 in the intestine of mice in the Con, IR, IR + GT groups. Each bar represents mean ± SD of each group of mice (n = 6). ∗*p* < 0.05.

We examined the effect of GT on ROS-derived oxidative stress following irradiation by measuring the gene expression of 8-hydroxy-2-deoxyguanosine (8-OHdG), a marker of ROS-induced DNA damage in tissues. Irradiation significantly increased 8-OHdG expression in the intestinal epithelium of the mice; however, GT treatment significantly decreased irradiation-induced 8-OHdG expression in the epithelial layer (Fig. 2A). Specifically, the mRNA levels of 8-OHdG was markedly lower in the IR + GT group than in the IR group. GT treatment increased Nrf2 expression both at the protein and mRNA levels (Fig. 2B and C). Additionally, the mRNA levels of Ho1 and Nqo1 were increased in the IR + GT mice than in the IR mice (Fig. 2C), indicating that GT treatment inhibited oxidative stress via NRF2 activation. As radiation-induced intestinal injury is characterized by inflammation with increased leukocyte infiltration and expression of proinflammatory cytokines, such as IL-6, TNF-a, and PAI1 in the intestine [26], we investigated the effects of GT on inflammatory response in the intestines of irradiated mice. The level of the macrophage marker CD68 corresponds with the degree of inflammation in intestinal diseases [27]. CD68-positive cells were higher in the intestinal mucosa of IR group compared with that of the control group (Fig. 3A); however, GT treatment inhibited radiation-induced increase in macrophage recruitment (Fig. 2D). IL-6 and TNF-a play critical roles in the inflammatory response and these cytokines are known to increase in acute radiation-induced intestinal damage [28]. PAI1 is an endothelial-derived inflammatory mediator expressed following irradiation [26]. Compared with that in the non-irradiated group, Il-6, Tnf-a, and Pai1 mRNA levels significantly increased in the intestinal tissues of irradiated mice (Fig. 2E). However, GT treatment suppressed radiation-induced increase in the mRNA expression of inflammatory cytokines (Fig. 3E), indicating that GT may reduce oxidative stress and inhibit intestinal inflammation in irradiated mice.

### 3.3 GT treatment reverses radiation-induced epithelial barrier damage in the intestine

To elucidate the effect of GT on the epithelial barrier function of irradiated mice, we examined the expression of junctional molecules and performed FITC-dextran absorption assay. Irradiation significantly increased the serum concentration of FITC, which was reduced by GT treatment (Fig. 3A). Additionally, GT treatment significantly reversed radiation-induced decrease in the expression of Villin (Fig. 3B and E). GT treatment reversed radiation-induced decrease in Zo1 and Cldn3 expression in the intestinal epithelium of the mice at both the mRNA and protein levels (Fig. 3C–E). Overall, these data suggest that GT treatment may ameliorate radiation-induced intestinal barrier damage with the recovery of TJs expression.

### 3.4 GT enhances epithelial barrier function in irradiated Caco2 monolayers via NRF2 pathway activation

**Fig. 4:**
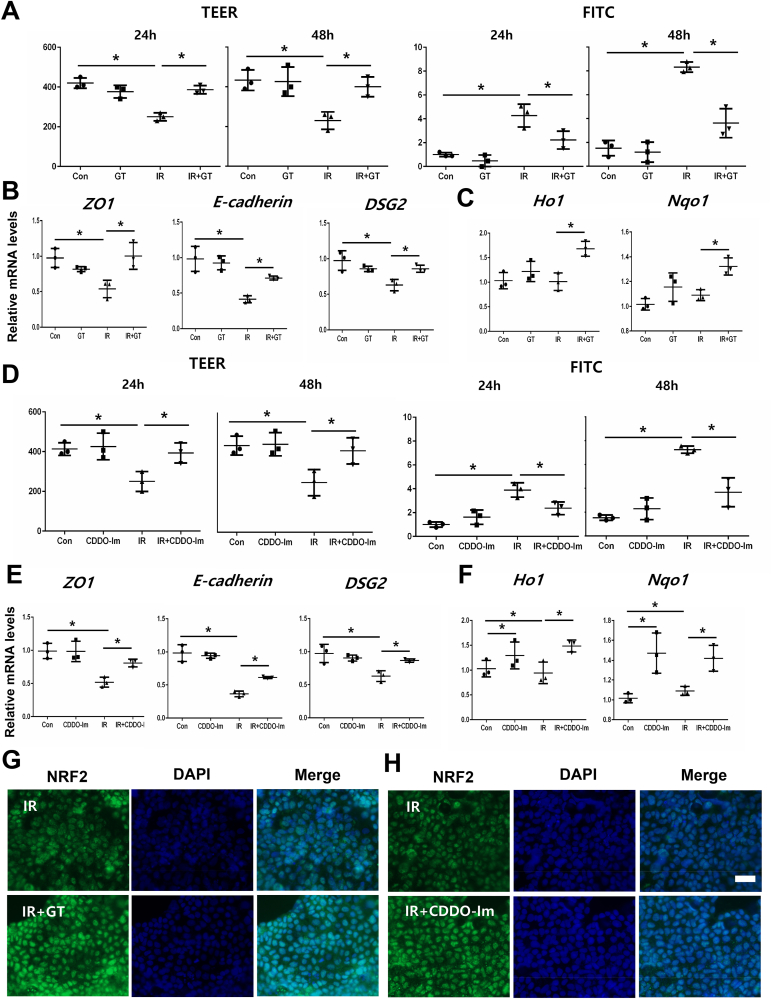
Gintonin (GT) reinforced barrier function in irradiated epithelial cells by activating NRF2 pathway**.** (A) TEER and FITC-dextran assay of Con, GT, IR, and IR + GT-treated Caco-2 cell monolayers. mRNA levels of (B) ZO1, E-cadherin, and DSG2, and (C) HO1, and NQO1. (D) TEER and FITC-dextran assay. mRNA levels of (E) ZO1, E-cadherin, and DSG2, and (F) NRF2-regulated genes, such as HO1 and NQO1 in Con, CDDO-Im, IR, IR + CDDO-Im-treated Caco-2 monolayers. (G) Immunofluorescence of NRF2 in GT or CDDO-Im treated irradiated Coco-2 cell monolayers. Bar = 40 μm. Each bar represents mean ± SD of each group (n = 3). ∗*p* < 0.05.

To examine whether GT directly affects paracellular permeability in irradiated epithelial cells, we performed TEER and 4-kDa FITC-dextran assays using a Caco-2 monolayer. Irradiated Caco-2 monolayers displayed hyper-permeability, which was consistent with a decrease in TEER levels and an increase in FITC concentrations in the lower chamber (Fig. 4A). In contrast, GT treatment significantly improved radiation-induced epithelial barrier dysfunction, as indicated by TEER and FITC-dextran levels (Fig. 4A). Additionally, GT treatment attenuated radiation-induced loss of intercellular junction expression (ZO1, E-cadherin, and DSG2) and increased nuclear translocation of NRF2 and NRF2-related gene expression in Caco-2 monolayers (Fig. 4B, C and 4G).

In the mouse experiment, GT activated the expression of NRF and NRF-regulated genes and enhanced the intestinal epithelial barrier in irradiated mice. Therefore, Caco-2 monolayers were treated with the NRF2 activator CDDO-lm to clarify whether NRF2 activation directly affects epithelial permeability during irradiation. CDDO-Im treatment increased NRF2 activation and the expression of NRF2-regulated genes, such as HO1 and NQO1, under non-irradiated and irradiated conditions (Fig. 4F and H). Additionally, CDDO-Im treatment significantly increased TEER levels, decreased the FITC concentrations, and increased the gene levels of intercellular junction molecules in irradiated Caco-2 monolayers (Fig. 4D).

To determine whether GT's action in protecting epithelial integrity is directly mediated by NRF2, we co-treated GT with an NRF2 inhibitor. ML385 treatment functionaly disrupted epithelial integrity with decrease NRF2 activation and NRF2-related genes in GT treated irradiated Caco-2 monolayers (Suppl Figs. 1A, 1C, and 1D). Additionally, ML385 treatment decreased the gene levels of intercellular junction molecules in GT-treated condition (Suppl Fig. 1B). These results suggest that GT-induced NRF2 activation may improve epithelial permeability by restoring junctional molecules in irradiated epithelial cells.

### 3.5 NRF2 activator relieves radiation-induced intestinal damage by suppressing inflammation and ameliorating epithelial damage

**Fig. 5:**
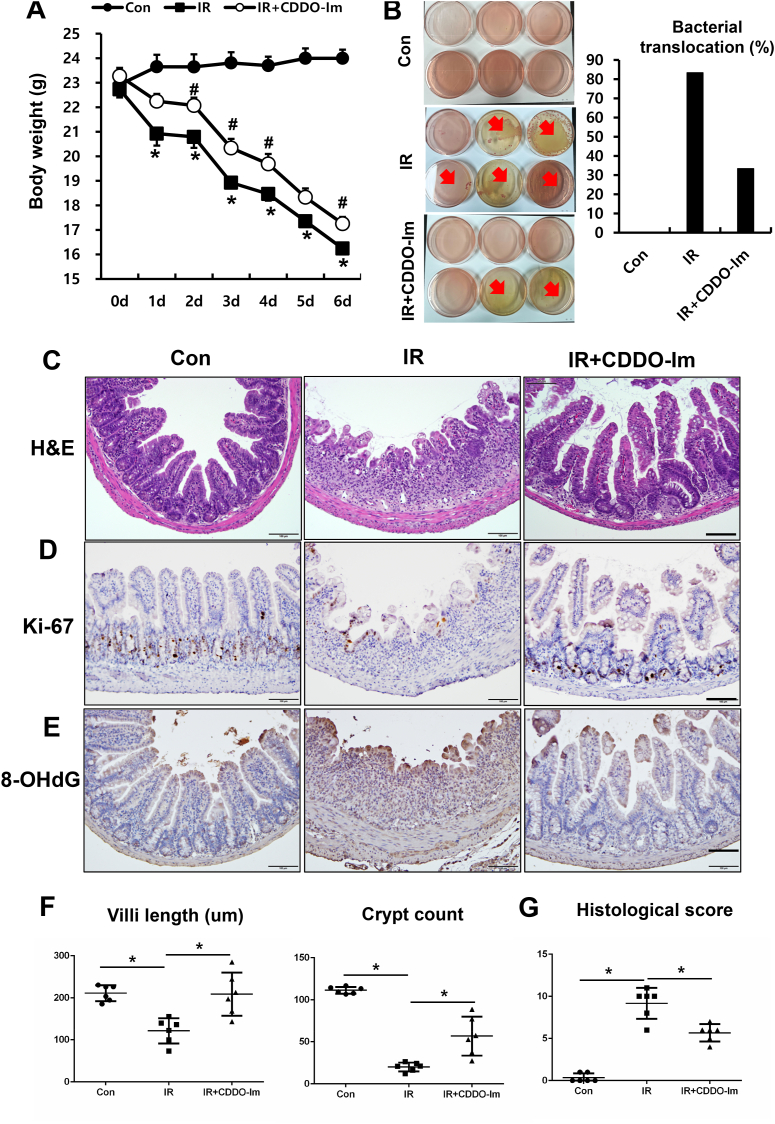
CDDO-Im treatment ameliorated radiation-induced intestinal damage. (A) Body weight and (B) the percentage of positive bacterial colonies in the lymph node of mice in the Con, IR, and IR + CDDO-Im groups. (C) H&E and immunohistochemistry for (D) Ki-67 and (E) 8-OHdG in the intestine. Bar = 100 μm. (F) Villi length and the number of crypt and (G) histological score of the intestine in the Con, CDDO-Im, IR, and IR + CDDO-Im groups. Each bar represents mean ± SD of each group of mice (n = 6). ∗*p* < 0.05.

**Fig. 6:**
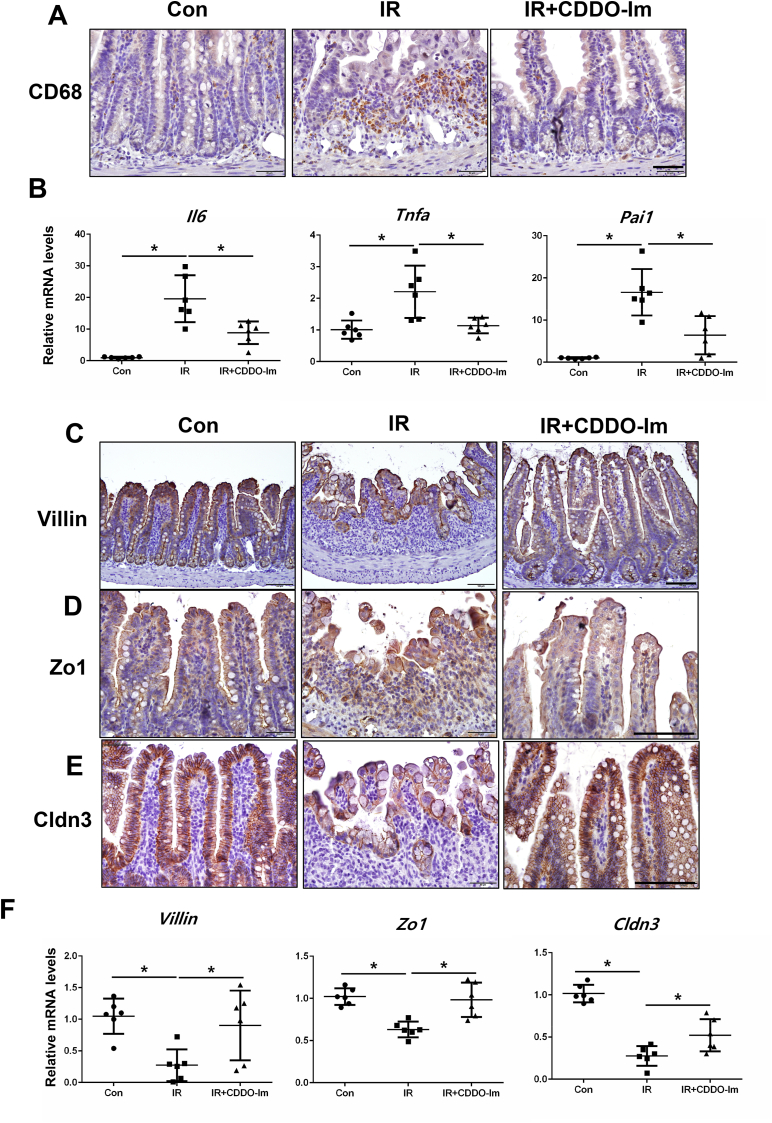
CDDO-Im treatment reduced inflammatory response and improved radiation-induced tight junction destruction in the irradiated intestine. (A) Immunostain of CD68 and (B) mRNA levels of inflammatory cytokines (Il6, Tnf-a, and Pai1) in the intestine of the Con, IR, and IR + CDDO-Im groups. Bar = 50 μm. Immunohistochemistry for (C) villin, (D) Zo1, and (E) Cldn3 expression. Bar = 100 μm. (F) mRNA levels of villin, Zo1, and Cldn3 in the intestine of the Con, IR, and IR + CDDO-Im groups. Each bar represents mean ± SD of each group of mice (n = 6). ∗*p* < 0.05.

To determine whether NRF2 could be a therapeutic target for radiation-induced intestinal injury, we intraperitoneally injected mice with CDDO-Im (1 mg/kg/day) daily after abdominal irradiation. Surprisingly, the body weights of the IR + CDDO-Im mice were markedly higher than those of the IR mice (Fig. 5A). Translocation of bacteria to other organs indicates severe injury to the intestinal epithelial barrier. Therefore, bacterial translocation assay was tested to elucidate the protective effect of NRF2 activator on irradiation-induced barrier damage using mesenteric lymph. The percentage of colony occurrence was markedly higher in the IR group than in the control group; however, CDDO-Im treatment decreased the percentage of positive bacterial colonies compared with that in the IR group (Fig. 5B). Additionally, CDDO-Im treatment relieved histological damage including villus lengths, crypt counts, and epithelial proliferative property compared with those in the IR group (Fig. 5C–G). Moreover, CDDO-Im treatment attenuated radiation-derived oxidative stress in the intestine (Fig. 5E), and inhibited inflammatory response by suppressing macrophage accumulation in the mucosal/submucosal layers and decreasing the inflammatory cytokines expression, such as Il6, Tnf-a, and Pai1 (Fig. 6A and B).

Furthermore, immunostaining indicated that CDDO-Im treatment ameliorated epithelial damage and the loss of intercellular junctions in the IR mice (Fig. 6C–E). Specifically, the mRNA levels of Villin, Zo1, and Cldn3 were significantly higher in the IR + CDDO-Im mice than in the IR mice (Fig. 6F). Overall, these results indicate that NRF2 activation regulates inflammatory response and epithelial integrity in radiation-induced intestinal injury.

## 4 Discussion

GI injury is a well-known acute radiation syndrome and is observed in patients undergoing radiation therapy. Radiation exposure can cause severe adverse effects in the GI tract, such as hemorrhage, loss of electrolytes and fluid, bacterial infection, and endotoxemia [29]. Some morphological characteristics of acute GI injury include the inhibition of epithelial cell proliferation and apoptotic cell death in the crypts of the intestine [2,29]. In mice, irradiation overdose of 10 Gy primarily lead to GI injury, resulting in diarrhea, dehydration, sepsis, intestinal hemorrhage, and death within 10–15 d post-exposure [30]. Studies have shown that one out of five patients receiving radiation therapy present with clinical signs of radiation-induced enteropathy, including obstruction, malabsorption, fistula, and/or other complications [31]. Additionally, 90 % of pelvic radiotherapy-received patients experience acute damage in GI tract after radiotherapy, and 50 % experience a significant reduction in their quality of life for longtime [32]. However, there are no therapeutics for radiation-induced GI toxicity in the clinical setting [33]. Therefore, studies are necessary to develop an effective agent for mitigating radiation-induced intestinal damage. In this study, we assessed the acute effects on the small intestine following radiation exposure induced by a 13.5 Gy dose [8,26,28]. This approach allowed us to explore its potential as a mitigator under radiotherapy-received patients and sub-lethal dose irradiated condition. We demonstrated the therapeutic effects of GT in a mouse model of acute radiation-induced intestinal injury. GT treatment-induced NRF2 activation inhibited irradiation-associated inflammation and enhanced epithelial barrier function. Therefore, GT could be used as a potential therapeutic approach for the treatment of radiation-induced toxicity and intestinal injury.

Panax ginseng is a well-known traditional herbal medicine and is considered an energizing herbal plant [34]. Panax ginseng contains several effective ingredients, such assteroidal saponins, protopanaxadiols, and protopanaxatriols, collectively known as ginsenosides. In the last few decades, ginseng and its several fractions have been shown to have antioxidative and anticancer effects; additionally, their ability to improve immunity and energy and combat diabetes mellitus, cardiovascular diseases, and neurological diseases has been studied in both animal and clinical research [35]. GT, a non-saponin fraction isolated from Panax ginseng [16], has been reported to exert therapeutic effects on neurodegenerative and inflammatory diseases [18,25,36]. Choi et al. reported that the anti-oxidative effects of GT are associated with NRF2 activation, which mediates protective effects against oxidative stress, apoptosis, and inflammation by regulating antioxidant enzymes, such as HO1 and NQO1 [18,25]. Additionally, NRF2 activation protects against chronic inflammatory diseases, such as multiple sclerosis, chronic kidney disease, and cardiovascular disease, by mitigating oxidative stress and inflammation [37]. As oxidative stress is a vital factor in the progression of radiation-induced damage, antioxidants are important for the development of therapeutic agents [38]. In radiation-induced intestinal injury, the nuclear translocation of NRF2 increases within the initial 48 h and gradually decreases thereafter, showing a similar pattern in the expression of NRF2-related genes [39]. Furthermore, it has been reported that the mRNA expressions of HO-1 and NQO-1 are reduced in intestinal epithelial cell lines exposed to radiation compared to normal condition [40]. In this study, we demonstrated that GT decreases radiation-induced oxidative stress in epithelial cells and alleviates acute intestinal damage by activating NRF2 pathway. Additionally, treatment with NRF2 activator alleviated radiation-induced intestinal injury.

Oxidative stress also induces the inflammatory response during radiation exposure. The anti-inflammatory effects of GT have been widely reported in neurodegenerative diseases and autoimmune diseases [25,36]. GT suppresses inflammation by inactivating nuclear factor-kappa B (NF-kB) signaling and mitogen-activated protein kinases in inflammatory cells [41,42]. NRF2 induces anti-inflammatory activity by inhibiting the transcription of proinflammatory cytokine genes or regulating NF-kB signaling [14,43]. In inflammatory bowel disease (IBD), NRF2 activation protects intestinal epithelial integrity by regulating inflammation and inducing the antioxidant system [15,44]. In the present study, GT treatment decreased the accumulation of inflammatory cells and significantly reduced the expression of inflammatory cytokines, such as Il6, Tnf-a, and Pai1, in the intestines of irradiated mice. Additionally, NRF2 activator treatment inhibited the accumulation of inflammatory cells and the inflammatory cytokines expression in radiation-induced intestinal injury. Overall, these findings suggest that GT inhibits intestinal inflammatory response in acute radiation-induced intestinal damage with NRF2 activation.

The intestinal epithelial layer plays an important role in resisting external environmental stimuli, and junctional proteins, especially TJs, control the paracellular permeability of the intestinal epithelium. Rao et al. demonstrated that oxidative stress disrupts intestinal epithelial TJs [9,45]. NRF2 has been reported to prevent epithelial barrier damage in various disease models, including *Salmonella typhimurium* infection [46], IBD [[46], [47], [48]], severe sepsis [49], and traumatic brain injury-induced intestinal mucosal damage [50]. Additionally, research has highlighted the significance of the NRF2 pathway in maintaining barrier integrity. A study using a colitis model demonstrated that an NRF2 activator reduced colon damage by enhancing the expression of barrier components. Moreover, in mice lacking NRF2, TNBS administration led to more severe barrier damage and heightened inflammatory responses [15].

Barrier functions, including intercellular junction expression, are sensitive to irradiation. Radiation exposure induces rapid TJ disruption and reorganization of the actin cytoskeleton in the intestinal epithelial cells within hours [51]. Ionizing radiation induces the loss of epithelial cell proliferation and results in increased intestinal permeability, including passage of intestinal bacteria, bacteremia, and exacerbation of mucosal inflammation within days [52,53]. CLDN3 and ZO1 are essential TJs that maintain the integrity of the epithelial barrier [54] and are significantly downregulated under radiation exposure, leading to increased gut permeability and systemic inflammatory responses [55]. In the present study, GT treatment recovered radiation-induced intestinal barrier dysfunction both of in vivo and in vitro models and improved the loss of Zo1 and Cldn3 expression in irradiated epithelial cells in a mouse model. In Caco-2 cell monolayers, GT reinforced intestinal barrier function by increasing the expression of junctional molecules, including TJs, with NRF2 activation. Additionally, treatment with an NRF2 activator attenuated radiation-induced intestinal injury by enhancing epithelial barrier function.

Conclusively, the results showed that GT inhibits inflammation and attenuates epithelial integrity in radiation-induced intestinal damage. Additionally, GT suppresses oxidative stress by increasing the expression of NRF2- and NRF2-regulated genes and reinforces intestinal epithelial barrier function in irradiated mice. Overall, GT protects against radiation-induced intestinal barrier damage by activating NRF2 and attenuating radiation-induced intestinal injury, suggesting a potential therapeutic role for GT in radiation-induced intestinal damage.

This study has several limitations, including the absence of direct animal experiments to confirm GT's role in inhibiting NRF2 activation, and a lack of understanding regarding the mechanism by which GT triggers NRF2 activation. Our experiments did not detect any alterations in LPA receptor 1 and 3 expression. This indicates that GT may activate NRF2 through an alternative pathway, although the immediate upstream factors remain unidentified in this research.

## Funding

This work was supported by a grant from the 10.13039/501100008003Korea Institute of Radiological and Medical Sciences (50531–2025; 50535–2025), which is funded by the Ministry of Science and Information and Communications Technology of the Korean government.

## Declaration of competing interest

The authors have no conflicting interests.
