# Supplementary material for: Gintonin enhances epithelial barrier function by activating NRF2 pathway in radiation-induced intestinal injury
Source: J Ginseng Res. 2025 Jan 19;49(3):248–59. doi: 10.1016/j.jgr.2025.01.003 (PMC12125585; doi:10.1016/j.jgr.2025.01.003)
Supplement: Multimedia component 1 [file mmc1.pptx]

## Slide 1
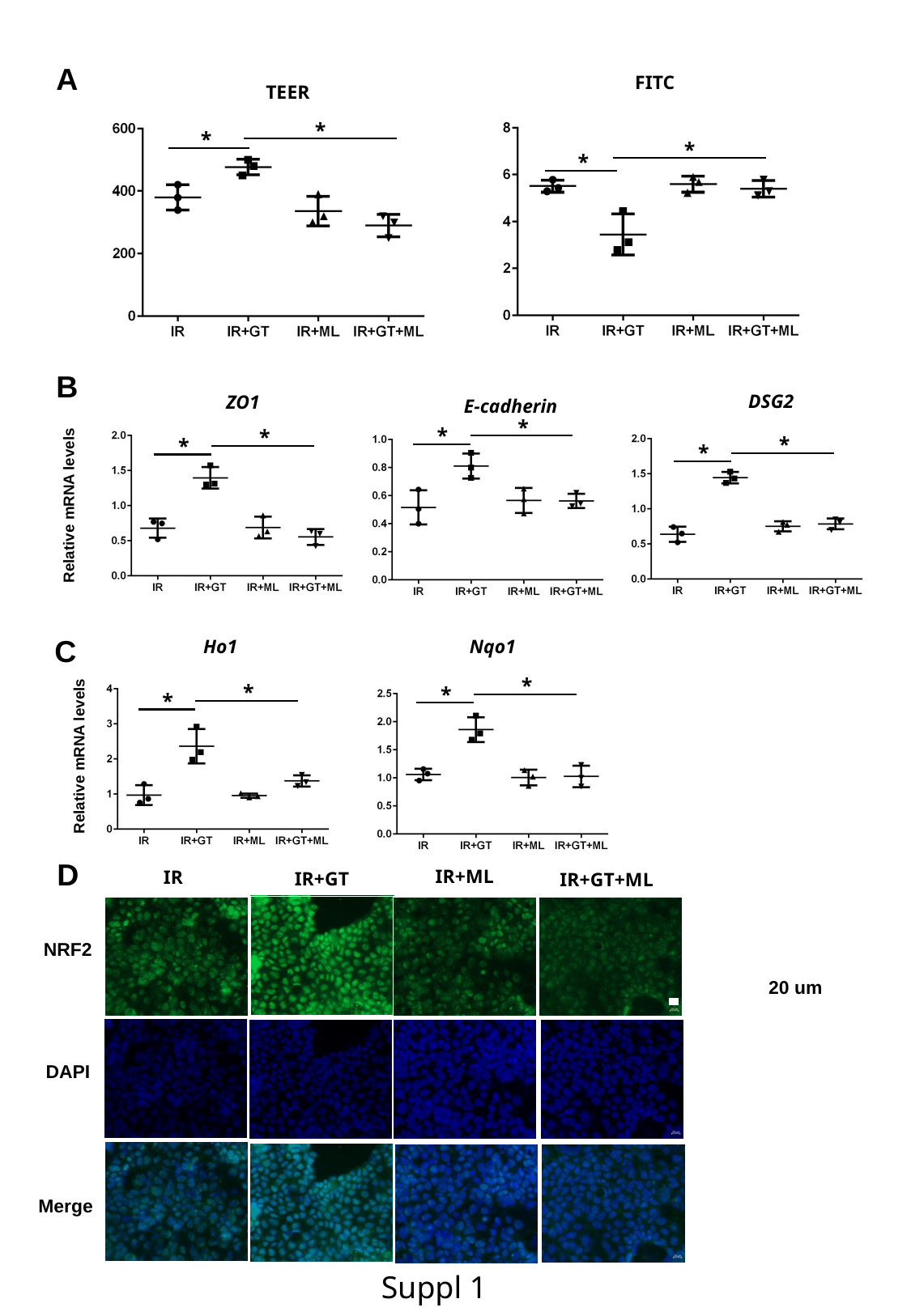

A
FITC
TEER
*
*
*
*
B
DSG2
ZO1
E-cadherin
Relative mRNA levels
*
*
*
*
*
*
C
Ho1
Nqo1
Relative mRNA levels
*
*
*
*
D
IR+ML
IR
IR+GT
IR+GT+ML
NRF2
20 um
DAPI
Merge
Suppl 1
